# Development and Validation of A Frusemide Card to Guide Self-Titration of Oral Frusemide in the Outpatient Setting

**DOI:** 10.21315/mjms-01-2026-068

**Published:** 2026-06-30

**Authors:** Nur Amalina Juhari, W Yus Haniff W Isa, Zurkurnai Yusof, Najib Majdi Yaacob, Siti Aisyah Hussin

**Affiliations:** 1 Department of Internal Medicine, School of Medical Sciences, Health Campus, Universiti Sains Malaysia, 16150 Kubang Kerian, Kota Bharu, Kelantan, Malaysia; 2 Cardiology Unit, Hospital Pakar Universiti Sains Malaysia, Universiti Sains Malaysia, Health Campus, Jalan Raja Perempuan Zainab 2, 16150 Kota Bharu, Kelantan, Malaysia; 3 Biostatistics and Research Methodology Unit, School of Medical Sciences, Health Campus, Universiti Sains Malaysia, 16150 Kubang Kerian, Kota Bharu, Kelantan, Malaysia

**Keywords:** diuretic titration, frusemide, heart failure, outpatient management, self-titration

## Abstract

**Background:**

Heart failure (HF) causes frequent hospitalisations due to fluid congestion. Patient-friendly self-titration tools are scarce. This study developed and validated a “Frusemide Card” to support safe outpatient dose adjustment.

**Methods:**

This cross-sectional development and validation study was conducted in two phases. Phase 1 involved developing the Frusemide Card through evidence-based content construction and graphical design. Phase 2 included content validation by six experts (four cardiologists, two pharmacists) using the Content Validity Index (CVI) and Content Validity Ratio (CVR), followed by face validation among 20 HF patients in two sequential cohorts using the Face Validity Index (FVI). Content validity included four domains: relevance, clarity, essentiality, and patient instructions. Face validation assessed clarity and comprehension to determine overall acceptance.

**Results:**

Expert validation showed perfect agreement for relevance and essentiality (I-CVI, CVR, and S-CVI/UA = 1.00). Clarity for domains 2–4 was acceptable (I-CVI ≥ 0.83), but domain 1 scored lower (I-CVI = 0.50), reducing the universal agreement (S-CVI/UA = 0.25), though average clarity was acceptable (S-CVI/Ave = 0.79). The first patient cohort reported good average clarity (S-FVI/Ave = 0.90) but low universal agreement (S-FVI/UA = 0.25). Following revisions based on feedback, the second patient cohort confirmed excellent clarity and acceptance, achieving perfect validity across all domains (I-FVI and S-FVI = 1.00).

**Conclusion:**

The Frusemide Card showed strong content and face validity, suggesting that it could be a useful tool for guiding the safe self-titration of oral frusemide in outpatient HF management.

## Introduction

Heart failure (HF) is a complex clinical syndrome characterised by structural or functional cardiac impairment that leads to inadequate cardiac output and organ perfusion. It remains a major public health problem, affecting over 64 million people globally and contributing substantially to morbidity, mortality, and healthcare costs (1, 2). In Malaysia, patients typically present 10 to 15 years earlier than in Western populations due to the high prevalence of hypertension, diabetes mellitus, and chronic kidney disease, which complicate disease progression and management (3).

Congestion and fluid retention are hallmarks of HF decompensation and account for most hospital admissions (3–5). Although triggers such as ischaemic heart disease, arrhythmia, infection, and valvular disorders are common, excessive salt or fluid intake contributes to about 9% to 10% of decompensation episodes and remains a modifiable risk factor (6–7). Therefore, early detection of weight gain and congestion is crucial, and patient empowerment through guided self-management may help prevent deterioration and reduce avoidable hospitalisations (8–10).

The cornerstone of symptomatic treatment is frusemide, a loop diuretic. However, both over- and under-diuresis carry risks such as hypotension, electrolyte disturbances, renal impairment, or persistent congestion (11, 12). Recent guidelines from the European Society of Cardiology (ESC) and the American College of Cardiology/American Heart Association/HF Society of America (ACC/AHA/HFSA) emphasise the need for personalised diuretic therapy adjustment to maintain a euvolemic status (4, 5). Both guidelines highlight the importance of patient education and self-care strategies, including daily weight monitoring and early recognition of fluid retention. However, these guidelines do not provide structured, patient-oriented tools to guide the self-adjustment of oral diuretics in an outpatient setting.

Despite these recommendations, there remains a gap between guideline-directed advice and practical implementation in daily clinical practice. Most patient education is delivered verbally or through general written materials, which may not provide sufficient structure for patients to adjust their diuretic therapy independently and safely. This limitation is particularly relevant in outpatient settings, where timely clinical review may not always be available. Therefore, there is a need for a simple, structured, and validated tool that translates clinical recommendations into actionable steps for patients.

Several studies have reported the benefits of flexible or patient-guided diuretic protocols, showing improved quality of life, exercise tolerance, and fewer readmissions (13–19). However, the existing protocols are institution-specific and have not undergone formal validation or adaptation for local use. To address this unmet need, we developed the Frusemide Card, a laminated, step-by-step visual guide designed to assist patients with stable HF in adjusting their oral frusemide dosage based on changes in body weight and blood pressure. A patient-instruction flyer accompanies this card and aims to promote self-care autonomy under clinician oversight.

The objective of this study is to develop and validate the Frusemide Card through content validation by a panel of clinical experts and face validation among patients with HF, ensuring the tool’s clinical relevance, clarity, and ease of use in outpatient care.

## Methods

### Study Design

A cross-sectional validation study incorporating both quantitative and qualitative feedback was conducted, as adapted from a previous study (20). This study was conducted in two distinct phases. Phase 1 involved the development of the Frusemide Card, which consisted of content construction and graphical design. Phase 2 involved systematic expert validation for content validity and patient assessments for face validity (Figure 1).

### Sample Size Calculation

The sample size for content and face validation was determined based on methodological recommendations for validation studies rather than a formal statistical power calculation. Previous literature suggests that a minimum of 5 to 10 experts is sufficient for content validation, while 10 participants are adequate for face validation to ensure reliable estimation of validity indices. Therefore, the inclusion of six experts and two cohorts of 10 patients each was considered appropriate for this study.

### Study Setting, Population, and Ethical Considerations

The study population consist of two specific groups: an expert panel and patient respondents. The expert panel consisted of six clinical experts, including four cardiologists and two clinical pharmacists, who were purposively selected based on their clinical experience, research expertise, and familiarity with patient education in HF. For face validation, 10 adult patients with HF from the HF Clinic at Hospital Pakar Universiti Sains Malaysia (HPUSM) were recruited using purposive sampling. Eligible patients were clinically stable, capable of understanding instructions, and willing to participate and provide meaningful feedback. Ethical approval was obtained from the Human Research Ethics Committee of Universiti Sains Malaysia (study protocol code: USM/JEPeM/ KK/24020151), and the study was conducted following the principles of the Declaration of Helsinki. Prior to participation, all participants provided written informed consent. The study procedures maintained strict confidentiality and anonymity of participant information. In addition, participants were informed of their right to withdraw from the study at any stage without repercussions.

### Sampling Method

Purposive sampling was employed for the following reasons: to ensure inclusion of participants with relevant clinical experience for content validity (experts) and adequate understanding of HF self-management for face validity (patients). There were no dropouts among the recruited participants, as all experts and patients completed the validation process and returned fully completed evaluation forms.

### Eligibility Criteria for the Content Validity Study

Four cardiologists and two pharmacists will be involved in this validation study. The criteria for experts include the following:

- Consultant cardiologist for at least three years
- Clinical pharmacists involved in the management of HF for at least three years

### Eligibility Study for Face Validation

Inclusion criteria:

- HF Clinic patients
- Aged more than 18 years

Exclusion criteria:

- Respondents who are illiterate
- Respondents who do not consent, are non-cooperative, or do not complete the questionnaire
- Respondents with severe cognitive impairment (e.g. dementia)

### Phase 1: Development of the Frusemide Card

The development phase comprised two sequential steps. The first step involved the construction of comprehensive content through a structured process that integrated evidence from clinical guidelines, existing literature, and expert consensus. An extensive review of current international and national HF guidelines, including the 2023 Malaysian Clinical Practice Guidelines, 2021 ESC, and 2022 ACC/AHA/ HFSA (3–5), was conducted to identify key recommendations related to patient self-care, fluid management, and diuretic dose adjustment in patients with stable HF.

The core components incorporated into the Frusemide Card, such as daily weight monitoring, blood pressure assessment, and guidance for temporary diuretic dose adjustment, were derived from these guidelines, which emphasise maintaining euvolemia, using the lowest effective diuretic dose, and recognising early signs of fluid retention. In addition, findings from previous studies on flexible and guided diuretic titration regimens informed the practical elements of patient engagement and home-based monitoring (13–17, 19). Subsequently, expert discussions were held with consultant cardiologists at the HPUSM. These experts reviewed the preliminary draft of the card, advised on clinically relevant parameters, and recommended the use of simplified terminology to enhance patient comprehension.

In the second step, the validated content was incorporated into the graphical design of the Frusemide Card. A concise, user-friendly table was created to outline stepwise frusemide dosing instructions based on clinical parameters. An accompanying patient flyer was also developed to further explain card usage, daily monitoring practices, and instructions for when to seek medical advice. Both the card and flyer were designed with emphasis on readability, visual clarity, and practicality for use in outpatient settings.

### Phase 2: Validation of the Frusemide Card

The validation phase included content validation by experts and face validation by patients with HF.

#### Content Validity

A panel of six experts systematically evaluated content validity through a structured validation process. Initially, a content validation form was prepared that clearly outlined domains (relevance, clarity, essentiality) and corresponding items, along with explicit scoring instructions. Then, the selected experts independently reviewed and rated each domain and item using evaluation forms. This validation study involved four cardiologists and two pharmacists. The criteria for selecting experts require that the individual must either be a consultant cardiologist with a minimum of three years’ experience or a clinical pharmacist who has been actively engaged in managing HF clinics for at least three years. To minimise potential bias, the experts were recruited from different institutions across Malaysia, including university hospitals and Ministry of Health hospitals. The cardiologists were from Hospital Al-Sultan Abdullah, Universiti Teknologi MARA and the National Heart Institute, while the pharmacists were from HPUSM and Hospital Serdang.

Each item was rated using two quantitative instruments: the Content Validity Index (CVI) and the Content Validity Ratio (CVR). The CVI employed a 4-point Likert scale to measure relevance and clarity (1 = not relevant/ clear; 4 = highly relevant/clear). Essentiality was measured by the CVR on a 3-point scale (essential, useful but not essential, or not necessary) (21–23). The experts also provided written qualitative feedback for each domain, recommending improvements as necessary.

For content validation, analyses included computations for the Item-level Content Validity Index (I-CVI), Scale-level Content Validity Index (S-CVI), Scale-level Content Validity Index/Average (S-CVI/Ave), Scale-level Content Validity Index/Universal Agreement (S-CVI/UA), Proportion of Chance Agreement (Pc) and Kappa statistics (κ). The CVI indices were calculated using these formulas:

- I-CVI = The total number of experts who rated an item as 3 or 4/The total number of experts
- S-CVI/Ave = Sum of I-CVI/Number of items in total
- S-CVI/UA = Number of items rated as relevant by all experts/Number of items in total

Since expert agreement can sometimes happen by chance, the probability of such chance agreement (Pc) for each item was determined using the binomial probability formula. To account for this, the Kappa statistic (κ) was employed as an index of inter-rater reliability, adjusting for the likelihood of agreement occurring randomly (23, 24).

#### Face Validity

Face validity focused on patient comprehension and acceptability and was evaluated through structured patient assessments. Initially, a simplified face validation form was prepared to evaluate clarity and comprehensibility. Ten patients were purposively recruited from the HF Clinic at HPUSM to participate in the face validity assessment. Patients who attended the HF Clinic with a diagnosis of HF (either reduced or preserved ejection fraction) and were receiving oral frusemide therapy, aged > 18 years, were included. Patients who were illiterate, did not provide consent, were non-cooperative, failed to complete the questionnaire, or had severe cognitive impairment such as dementia were excluded.

Participants reviewed the Frusemide Card and accompanying flyer, scoring each domain item on a 4-point Likert scale (1 = unclear/incomprehensible; 4 = very clear/ comprehensible) (25, 26). Patient comments and suggestions were documented, reviewed, and incorporated into revisions to improve the tool. The raw scores were calculated for the item-level Face Validity Index (I-FVI) and the scale-level Face Validity Index (S-FVI) for each comprehensibility and clarity, employing the scale-level average method (S-FVI/Ave) and universal agreement method (S-FVI/UA). Prior to the calculation of the FVI, the clarity and comprehension ratings were recoded as 1 (a scale of 3 or 4) or 0 (a scale of 1 or 2). The acceptable cut-off score of FVI is at least 0.83 for 10 raters. The formulas of the FVI indices are as follows:

- I-FVI = (agreed item)/(number of raters)
- S-FVI/Ave = (sum of I-FVI scores)/ (number of items)
- S-FVI/UA = (sum of UA scores)/ (number of items) (26).

### Statistical Analysis

All statistical analyses were descriptive. Demographic characteristics were analysed using IBM SPSS Statistics version 30 (IBM^®^, Armonk, New York, United States). The content and face validation calculations were executed in Microsoft Excel (Microsoft Corporation, United States). Qualitative feedback was obtained from experts in the form of descriptive written comments, which were used to identify areas for improvement and guide the iterative refinement of the Frusemide Card. No formal qualitative analysis was conducted.

## Results

### Phase 1: Development of the Frusemide Card

In Phase 1, the Frusemide Card was developed using a structured, evidence-based method. The card was designed as a laminated, table-formatted guide with an additional patient-instruction flyer. The content was derived from international HF guidelines and supported by local clinical practice references (Figure 2).

Dose adjustment parameters included daily body weight difference from dry weight and systolic blood pressure, selected for practicality in routine self-monitoring and their established relevance in detecting early congestion or hypotension. The Frusemide Card was structured as a stepwise dosing table, allowing patients to identify the appropriate frusemide dose adjustment based on measured weight changes and blood pressure readings. A threshold of 1.5 kg above dry weight was adopted, consistent with guideline recommendations, to optimise sensitivity for early fluid retention while minimising inappropriate dose escalation.

An additional patient education flyer was produced to complement the Frusemide Card. The flyer contained illustrated instructions for the accurate measurement and documentation of blood pressure and body weight, as well as guidance for dose adjustment. Safety information was also included, advising patients to seek medical attention if weight change exceeded guideline thresholds or if systolic blood pressure fell below 100 mmHg.

### Phase 2: Validation of the Frusemide Card

Phase 2 validation comprised expert content review followed by patient face validation of the Frusemide Card.

#### Content Validity

The experts from multiple institutions with backgrounds in diuretic titration, patient education, and outpatient HF management conducted the content validity assessment. The experts were invited via email, which contained a study overview, the Frusemide Card prototype, and standardised evaluation forms. The assessments were completed independently, without the influence of the researcher or other expert panel members. This approach ensured objective evaluation of item relevance, clarity, essentiality, and qualitative feedback, as well as reduced the risk of bias.

All four domains demonstrated excellent agreement among experts regarding their relevance and essentiality, achieving perfect scores (I-CVI and CVR = 1.00), thereby meeting the predefined acceptable thresholds (I-CVI ≥ 0.83, CVR ≥ 0.99) (Table 1 and Table 2). In contrast, the clarity assessment revealed variability across domains. Domain 1 (difference in body weight compared to dry weight [kg]) obtained an I-CVI of 0.50 with a Kappa of 0.27, indicating poor agreement and highlighting the need for revision. Domain 2 (systolic blood pressure [mmHg]) scored an I-CVI of 1.00 and a Kappa of 1.00, indicating excellent agreement. Domains 3 and 4 (dose adjustment and patient instructions) achieved I-CVI values of 0.83, with a Kappa value of 0.82 indicating good-to-excellent agreement (Table 1).

At the scale-level, the average S-CVI/ Ave and universal agreement S-CVA/UA for relevancy were both 1.00. Meanwhile, the average S-CVI/Ave for clarity was 0.79, which is considered acceptable, although the universal agreement S-CVI/UA was only 0.25 due to the lower clarity rating of domain 1 (Table 1). The incorporation of Kappa statistics strengthened the interpretation of content validity by adjusting for chance agreement. Domains with I-CVI values at or above the cut-off (≥ 0.83) generally corresponded to good-to-excellent Kappa values (κ ≥ 0.74), reflecting robust inter-rater reliability. The only exception was domain 1 (body weight difference), where the wording or presentation led to lower clarity scores despite expert recognition of its clinical importance. Consequently, domain 1 was revised based on expert recommendations, enhancing its clarity before progressing to the next face validation phase.

#### Face Validity

Face validity was assessed in two rounds, each with 10 patients with HF at HPUSM. For the first cohort of patients with HF (*n* = 10), the mean age was 54.6 (16.23) years, with 60% male and 40% female participants. The mean ejection fraction (EF) was 41.1 (19.96%), indicating a mixed representation of patients with both preserved and reduced systolic function. In terms of employment background, 40% were housewives, 40% were unemployed, 10% were pensioners, and 10% were non-pensioner retirees. The mean age of the second cohort of patients with HF (*n* = 10) was 58.0 (11.02) years, with a similar gender distribution (60% male, 40% female). The mean EF was 37.3 (16.60%). Regarding employment, 30% were retirees, 20% were housewives, and 20% were pensioners, while 10% were employed in government, private, and self-employed sectors. Overall, both cohorts included a balanced gender distribution and a range of HF severities and occupational backgrounds, reflecting a reasonable representation of typical patients with HF attending outpatient clinics.

In the first face validation round, the scale-level Face Validity Index average (S-FVI/ Ave) for clarity and comprehension was 0.90, indicating good overall acceptability. However, the scale-level universal agreement (S-FVI/UA) was notably low at 0.25, reflecting significant variability in patient perceptions regarding the clarity and comprehensibility of certain instructions (Table 3). Qualitative feedback highlighted specific areas that required simplification and improved readability. Based on this patient feedback, targeted revisions were performed to address these issues.

Following these revisions, a second face validity evaluation was conducted with a new cohort of 10 patients with HF. The outcomes were substantially improved, with all four domains achieving perfect item-level scores (I-FVI = 1.00) (Table 3). The scale-level indices for both average clarity and comprehension (S-FVI/Ave) and universal agreement (S-FVI/ UA) reached a maximum score of 1.00, indicating unanimous patient agreement regarding the clarity, ease of use, and practicality of the revised Frusemide Card. Patients reported that the final tool was intuitive, clearly presented, and highly beneficial for supporting their daily self-management.

## Discussion

This study aimed to develop and validate a Frusemide Card as a structured, patient-centred tool to guide the self-titration of oral frusemide in outpatient settings. Despite the high prevalence of HF and the central role of diuretics in its management, there remains a lack of validated patient-centred tools to guide safe and effective dose adjustments at home (1, 2, 14, 15, 27, 28). Most existing strategies rely on general advice delivered during clinic visits, leaving patients without structured day-to-day management guidance (3–5). This gap increases the risk of both under-diuresis, with resultant fluid congestion, and over-diuresis, with the potential for hypovolemia, hypotension, or renal dysfunction. The Frusemide Card provides a practical solution that can be used to translate complex clinical recommendations into clear, actionable steps that patients can implement at home. The card supports structured and guided diuretic self-management as part of routine medical follow-up by combining weight and blood pressure monitoring with simplified dose adjustment instructions.

Weight monitoring is the cornerstone of outpatient HF management. Previous studies have demonstrated that changes in daily weight provide an early indicator of fluid status, allowing timely diuretic adjustment and potentially preventing hospital admissions (11, 12, 17, 29). The development of the Frusemide Card incorporated evidence-based thresholds derived from international guidelines. The 2021 ESC guidelines recommend using diuretics to achieve and maintain euvolemia at the minimum effective dose, with effective diuresis typically reflected by a daily weight reduction of 0.75 to 1.0 kg. Furthermore, the ESC guidelines highlight that a weight gain of ≥ 2 kg within two to three days should prompt urgent medical review (4). In parallel, the 2022 AHA/ACC/ HFSA guidelines emphasise self-monitoring and recommend medical consultation if patients experience weight gain of more than two to three pounds (0.9 to 1.3 kg) in 24 h, particularly if worsening symptoms accompany the weight gain (5). The Frusemide Card adopted a cut-off of 1.5 kg above dry weight as the threshold for dose adjustment to balance these recommendations. This threshold represents a clinically meaningful increase in weight, signalling fluid retention, while being conservative enough to trigger intervention before the progress of decompensation. In this way, the chosen cut-off harmonises international recommendations and translates them into a simplified metric for patient use, enhancing both safety and usability.

The development of this card was guided by evidence from existing studies that explored flexible or guided diuretic regimens in HF management. A previous study demonstrated that a structured sliding-scale diuretic titration protocol improved patients’ exercise tolerance and quality of life and reduced emergency visits without increasing adverse events (13). Similarly, another study implemented a home-based diuretic protocol supported by home health services, which resulted in fewer readmissions (14), while another study reported that a diuretic adjustment algorithm reduced hospitalisations in a randomised trial (15). These studies, along with recommendations from the 2021 ESC and 2022 AHA/ACC/HFSA guidelines, highlight that patient education and structured diuretic adjustment under supervision can improve outcomes (4, 5). Validation is an essential step to ensure that the tool is both evidence-based and practical for patient use. Even well-designed tools may fail without validation due to misinterpretation or lack of acceptability (21, 22). The content validation confirmed that the Frusemide Card was aligned with current HF guidelines and was considered relevant by experts (23). Face validation, which involves patients directly, provided equally important insights into clarity, comprehension, and usability in real-world contexts (25, 26). By integrating both expert and patient perspectives, the study ensured that the final version of the card is scientifically robust and user-friendly.

The Frusemide Card demonstrated excellent overall content validity, with indices surpassing acceptable thresholds for both relevance and essentiality. Nevertheless, domain 1 (Column for “Difference in body weight compared to dry weight [kg]”) was initially rated lower in clarity by three out of six experts, resulting in an item-level CVI of 0.50. Their feedback indicated the need for improved phrasing and presentation, which was subsequently revised. Previous instrument development studies have reported similar observations, where clinically important items may initially demonstrate lower clarity due to complexity in translating medical concepts into patient-friendly language (21, 22). In contrast, the domains addressing systolic blood pressure, morning frusemide dose adjustment, and the instructional flyer achieved acceptable clarity and relevance from the outset.

Face validity findings highlighted the nuances of patient comprehension. The first cohort achieved a strong average face validity score (S-FVI/Ave = 0.90) but low universal agreement (S-FVI/UA = 0.25). Domains 3 and 4, which relate to dose adjustment and patient instructions, were the most challenging. The patients specifically noted that the flyer was “too packed and wordy,” and two patients struggled with the clarity of the dose adjustment instructions. These findings are consistent with previous studies, demonstrating that excessive information density and complex instructions can negatively impact comprehension and adherence, particularly among patients with varying levels of health literacy (8–10, 30, 31). The results also highlight the importance of tailoring patient education tools to diverse literacy levels and cognitive preferences. Revisions based on this feedback included simplifying the language, breaking down the instructions into shorter steps, and improving visual clarity. Recalling the initial patient cohort for reassessment was not feasible due to logistical constraints. Thus, a second patient cohort evaluated the revised tool, showing marked improvement with excellent comprehension, acceptance, and usability. Using a fresh cohort is methodologically advantageous when evaluating revised materials, as it mitigates potential bias from prior exposure and enhances the generalizability of the findings, ensuring broader applicability of the final Frusemide Card.

Patient input was invaluable in shaping the final version of the Frusemide Card. Beyond quantitative scoring, qualitative feedback highlighted areas for refinement that would not have been captured by expert review alone. The key themes were simplification of language, reduction of information density, and a stepwise instructional approach. Incorporating these suggestions ensured that the tool aligns with health literacy principles and is more likely to be accepted and used consistently by patients (25).

When compared with existing resources, several tools and protocols have previously been developed internationally to assist in the adjustment of diuretic therapy among patients with HF. Notably, the Agency for Clinical Innovation (ACI) Flexible Diuretic Regimen Clinical Practice Guideline provides a stepwise framework for oral furosemide titration. This guideline emphasises individualised adjustments based on daily weight, blood pressure, renal function, and symptoms (32). Although it is comprehensive, its narrative format requires substantial clinical judgement and may be less accessible to patients who are expected to interpret and implement the advice independently. A patient-directed extension of this guideline, the ACI Flexible Diuretic Regimen Action Plan, simplifies the recommendations (33). This approach empowers patients to recognise the early signs of congestion and to take appropriate action, such as increasing diuretic doses or seeking medical attention. However, although the presentation was patient-friendly, the Action Plan was not subjected to a formal validation process to ensure content accuracy and usability from both clinician and patient perspectives. Another example is the Furosemide Sliding Scale, developed by RxFiles Academic Detailing (part of the College of Pharmacy and Nutrition at the University of Saskatchewan). This tool provides specific numerical dose adjustments according to exact weight changes (for example, an increase of 1 kg corresponds to an additional 20 mg of furosemide) (34). Although highly precise, the sliding scale requires good numeracy and close clinical supervision, limiting its applicability in settings where patients may have lower health literacy or limited follow-up resources.

Patient comprehension and adherence are critical components of successful self-care. Studies have shown that patients’ understanding of their disease, literacy level, and self-efficacy significantly affect HF treatment plan adherence (8–10, 30). Tools that are visually clear, written in simple language, and reinforced by verbal counselling improve comprehension and self-management behaviours (31). During the face validity assessment, patients rated the card highly for clarity and comprehension, suggesting that the design was appropriate for its educational purpose. However, actual adherence and behavioural outcomes were not measured and should be addressed in future studies.

Although this study focused primarily on establishing the content and face validity of the Frusemide Card, the question of clinical safety is important when considering patient-led diuretic adjustment. Evidence from previous studies has suggested that self-adjustment can be safe and effective when implemented within structured, clinician-supervised programmes. For example, previous studies demonstrated that guided home-based diuretic protocols reduced emergency visits and hospitalisations without increasing the incidence of adverse events (13, 15). Similarly, the 2021 ESC guidelines state that patients with stable chronic HF may be educated to alter their diuretic dose based on weight and symptoms, provided they remain under regular clinical review (4). Future clinical validation studies are necessary to confirm its safety and real-world impact on patient outcomes. It is also important to acknowledge that this validation study did not establish clinical safety. The Frusemide Card is intended to support self-care supervised by clinicians, not to replace medical consultation. The 2021 ESC and 2022 AHA/ ACC/HFSA guidelines emphasise that patient self-adjustment should occur within structured follow-up programmes to ensure monitoring and safety (4, 5). Therefore, while the card demonstrated strong content and face validity, it should be considered a prototype pending further validation that includes the assessment of safety, usability in real-world settings, and impact on hospital readmissions.

### Strengths, Limitations, and Recommendations

A notable strength of this study lies in its rigorous, two-stage validation process involving both clinical experts and end-users. The iterative approach, informed by detailed expert and patient feedback, significantly improved the clarity, usability, and overall acceptability of the Frusemide Card.

However, this study has several limitations. The validation process involved a relatively small sample of 10 patients from a single tertiary centre, which may limit the generalizability of the findings to broader populations with different literacy levels or healthcare settings. Furthermore, this study evaluated only the content and face validity of the Frusemide Card, without assessing its clinical safety or real-world effectiveness, and only applied to oral frusemide, not other diuretics.

Future studies should validate the use of the Frusemide Card using criterion and construct measures to confirm its reliability and clinical utility. Larger multicenter or randomised trials are needed to assess its impact on adherence, self-care, symptoms, quality of life, and hospital readmissions, providing stronger evidence for integration into HF education and outpatient care.

## Conclusion

This study successfully developed and validated the Frusemide Card as a well-structured and patient-centred tool to safely guide the self-titration of oral frusemide in the outpatient management of HF. The findings demonstrated strong content and face validity, confirming that the tool is clear, relevant, and user-friendly for HF patients. These results align with the study objective and support its potential role in enhancing patient self-management. Although it shows promise as a supportive tool, further clinical studies are required to evaluate its safety, effectiveness, and impact on clinical outcomes before its implementation in routine clinical practice.

## Figures and Tables

**Figure 1:**
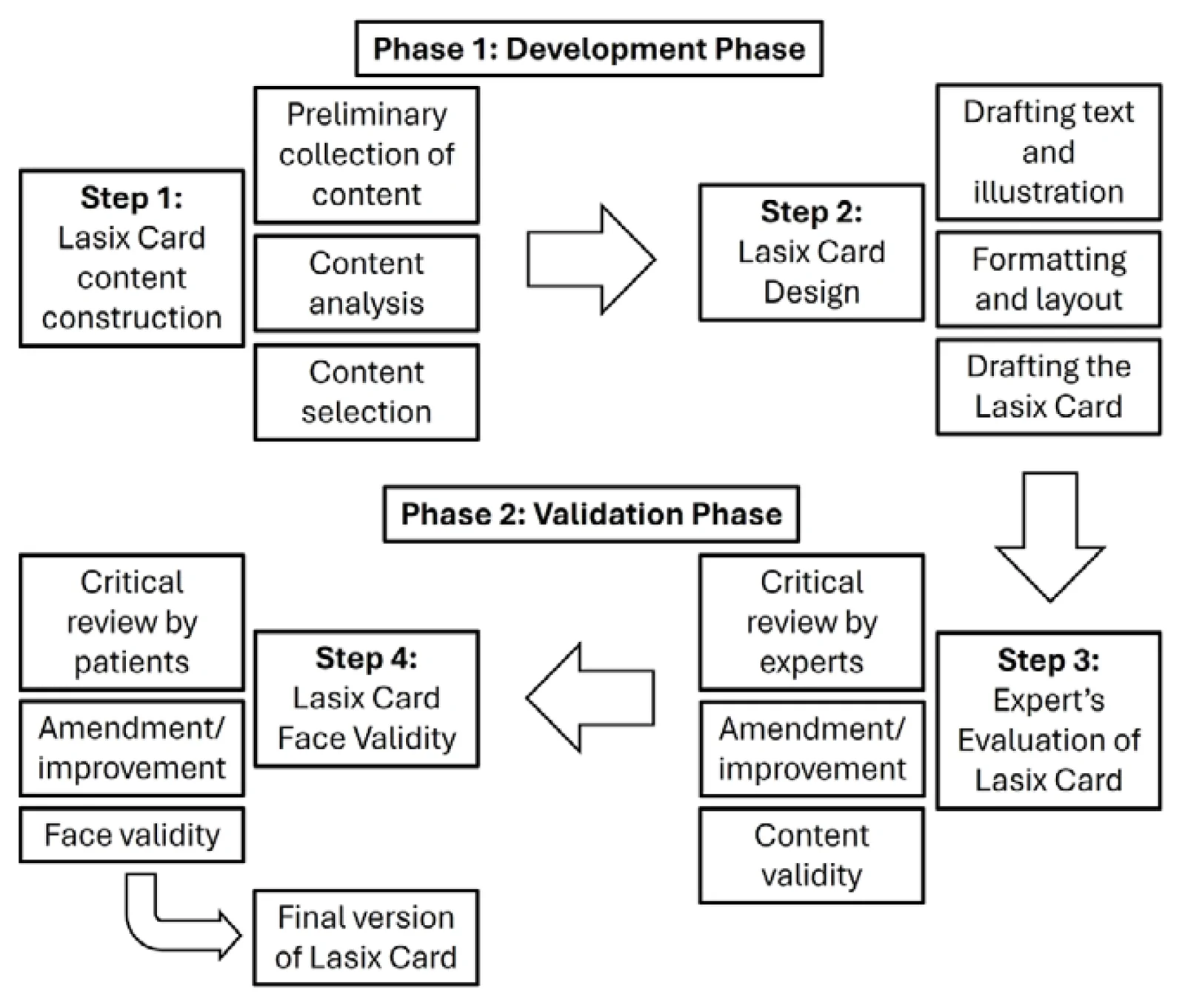
Process of development and validation, adapted from Farizan et al. (20).

**Figure 2:**
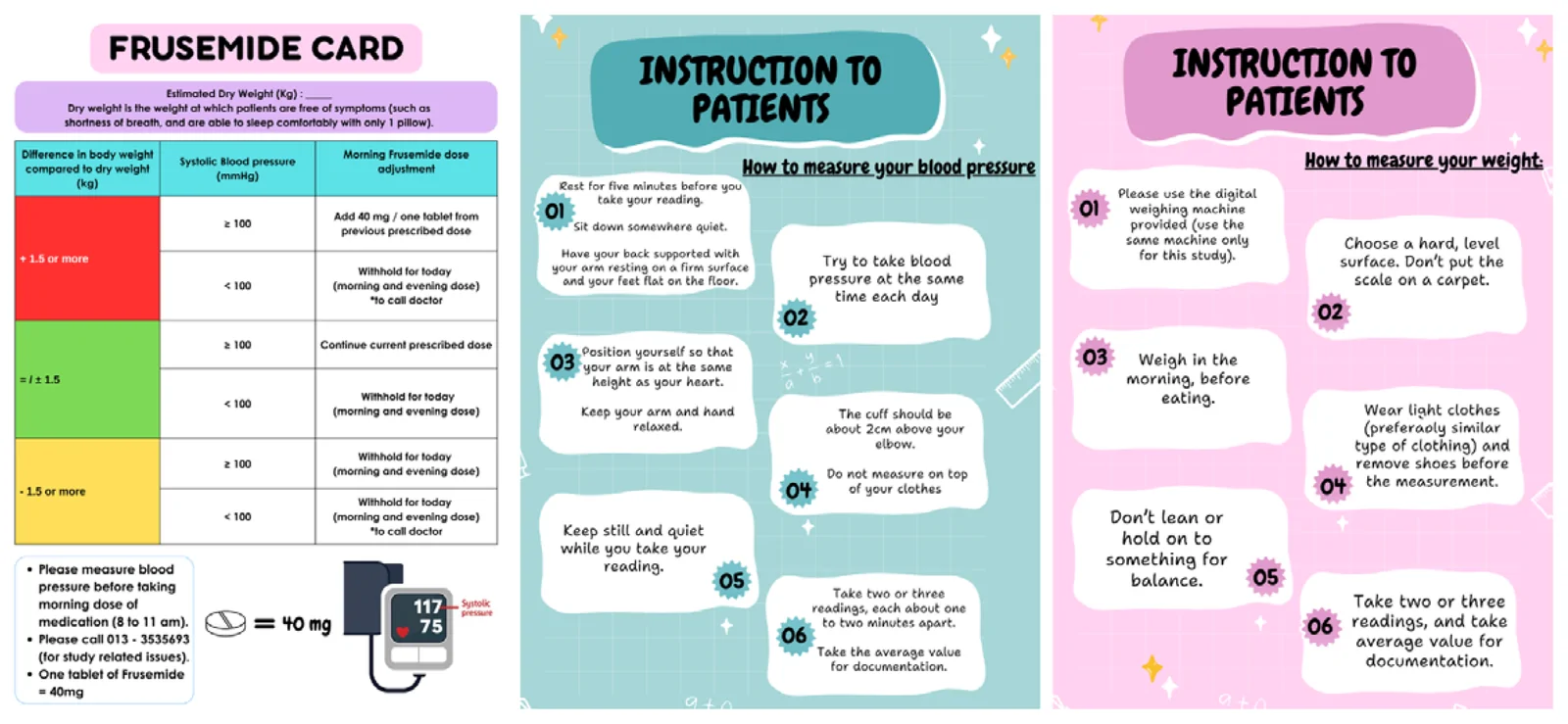
Frusemide Card and instructions for blood pressure and weight measurement.

**Table 1:** Assessment for the content validity (relevancy and clarity) of the four domains of the Frusemide Card by six experts using CVI.

Relevancy												
Domains	Expert 1	Expert 2	Expert 3	Expert 4	Expert 5	Expert 6	No. in Agreement	I-CVI	Pc	Kappa statistic	S-CVI/Ave	S-CVI/UA
1	√	√	√	√	√	√	6	1.00	0.016	1.00	1.00	1.00
2	√	√	√	√	√	√	6	1.00	0.016	1.00		
3	√	√	√	√	√	√	6	1.00	0.016	1.00		
4	√	√	√	√	√	√	6	1.00	0.016	1.00		
**Clarity**												
**Domains**	**Expert 1**	**Expert 2**	**Expert 3**	**Expert 4**	**Expert 5**	**Expert 6**	**No. in Agreement**	**I-CVI**	**Pc**	**Kappa statistic**	**S-CVI/Ave**	**S-CVI/UA**
1	√	√	-	-	-	√	3	0.50	0.313	0.27	0.79	0.25
2	√	√	√	√	√	√	6	1.00	0.016	1.00		
3	√	√	√	-	√	√	5	0.83	0.094	0.82		
4	√	√	-	√	√	√	5	0.83	0.094	0.82		

Domains rated as 3 or 4 on a 4-point scale; Domain 1 = difference in body weight (kg); Domain 2 = systolic blood pressure (mmHg); Domain 3 = dose adjustment; Domain 4 = patient instruction; I-CVI = Item-level Content Validity Index; Pc = Proportion of chance agreement; S-CVI/Ave = scale-level Content Validity Index average; S-CVI/UA = scale-level Content Validity Index universal agreement; S-CVI/Ave and S-CVI/UA for relevancy were both acceptable (1.00). For clarity, only S-CVI/Ave was acceptable (0.79), while S-CVI/UA was not acceptable (0.25) due to a lower clarity rating of domain 1; Domain 1 was revised for better clarity.

**Table 2:** Assessment of the content validity (essentiality) of the four domains of the Frusemide Card by six experts using CVR.

Domains	Expert 1	Expert 2	Expert 3	Expert 4	Expert 5	Expert 6	Ne	CVR
1	√	√	√	√	√	√	6.00	1.00
2	√	√	√	√	√	√	6.00	1.00
3	√	√	√	√	√	√	6.00	1.00
4	√	√	√	√	√	√	6.00	1.00

Domain 1 = difference in body weight (kg); Domain 2 = systolic blood pressure (mmHg); Domain 3 = dose adjustment; Domain 4 = patient instruction; Ne = Number of raters who rate the domain as essential; CVR: Content Validity Ratio; All domains showed excellent agreement for essentiality (CVR = 1.00).

**Table 3:** Assessment of face validity (clarity and comprehension) of four domains of the Frusemide Card by 10 patients (first and second set) using FVI

First set (*n* = 10)						Second set (*n* = 10)					
Clarity											
Domains	Raters in agreement	I-FVI	UA	S-FVI/Ave	S-FVI/UA	Domains	Raters in agreement	I-FVI	UA	S-FVI/Ave	S-FVI/UA
1	9	0.9	0	0.9	0.25	1	10	1.0	1	1.0	1.0
2	10	1.0	1			2	10	1.0	1		
3	8	0.8	0			3	10	1.0	1		
4	9	0.9	0			4	10	1.0	1		
**Comprehension**											
**Domains**	**Raters in agreement**	**I-FVI**	**UA**	**S-FVI/Ave**	**S-FVI/UA**	**Domains**	**Raters in agreement**	**I-FVI**	**UA**	**S-FVI/Ave**	**S-FVI/UA**
1	10	1.0	1	0.9	0.25	1	10	1.0	1	1.0	1.0
2	9	0.9	0			2	10	1.0	1		
3	9	0.9	0			3	10	1.0	1		
4	8	0.8	0			4	10	1.0	1		

Domains rated as 3 or 4 on a 4-point scale; Domain 1 = difference in body weight (kg); Domain 2 = systolic blood pressure (mmHg); Domain 3 = dose adjustment; Domain 4 = patient instruction; I-FVI = item-level Face Validity Index; UA = universal agreement; S-FVI/Ave = scale-level Face Validity Index average; S-FVI/UA = scale-level Face Validity Index universal agreement; All domains demonstrated improved clarity and comprehension following the revision, as reflected by the maximum I-FVI, S-FVI/Ave and S-FVI/UA score (1.0)
